# Feeding adaptation of François' langurs (*Trachypithecus francoisi*) to the fragmented limestone habitats in Southwest China

**DOI:** 10.1002/ece3.11269

**Published:** 2024-04-22

**Authors:** Wei Yao, Cheng‐Ming Huang, Jia‐Xin Zhao, Rong Huang, Wen‐Hua Li, Peng‐Lai Fan, Qi‐Hai Zhou

**Affiliations:** ^1^ Key Laboratory of Ecology of Rare and Endangered Species and Environmental Protection, Ministry of Education, Guangxi Key Laboratory of Rare and Endangered Animal Ecology, The Chongzuo White‐Headed Langur Field Observation and Research Station of Guangxi Guangxi Normal University Guilin China; ^2^ Institute of Zoology Chinese Academy of Sciences Beijing China; ^3^ Encheng National Nature Reserve Chongzuo China

**Keywords:** feeding adaptation, fragmentation, François' langurs, limestone habitat

## Abstract

Limestone forests are an unusual habitat for primates, especially fragmented limestone habitats. However, while some research has been conducted on François' langurs (*Trachypithecus francois*) in these habitats, there is still a need to improve the understanding of their behavioral adaptations to the fragmented limestone habitat. We collected data on the diet of François' langurs in a fragmented limestone habitat in Encheng National Nature Reserve, southwestern Guangxi, China using instantaneous scanning sampling, and their feeding adaptations to the fragmented forest were examined. The results indicated that a total of 101 species of plants were consumed by the langurs. They also fed on two non‐plant components, including cliff minerals and at least one species of insect. The langurs ate a higher number of food species in Encheng when compared with the other geographic populations, and they maintained a high level of food diversity and ate more vines. Moreover, they were highly selective in their use of vegetation in their home range, and fewer plants provided a high‐quality food source. During the season when food resources were scarce, the consumption of fruits and young leaves decreased as their availability decreased. This led to the use of other food components, such as mature leaves and seeds. The findings support that François' langurs adjust their feeding behavior to cope with seasonal and micro‐variations in their dietary requirements and to adapt to their particular environment.

## Graphical abstract

François' langurs in Encheng National Nature Reserve ate a higher number of food species in Encheng when compared with the other geographic populations, and they maintained a high level of food diversity and ate more vines. Moreover, they were highly selective in their use of vegetation in their home range. The findings support that François' langurs adjust their feeding behavior to cope with seasonal and micro‐variations in their dietary requirements and to adapt to their particular environment.

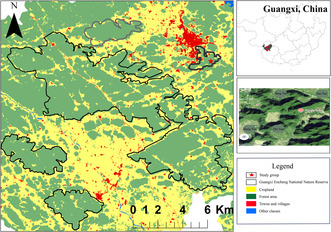

## 1 INTRODUCTION

Habitat loss and fragmentation are two of the biggest threats to the survival of non‐human primates (Crooks et al., 2017). High forest dependence makes primates vulnerable to deforestation and habitat fragmentation, and these threats increasingly force them to inhabit isolated small forest patches that are surrounded by anthropogenic activities. Consequently, populations have declined dramatically, with non‐human primates being one of the most vulnerable groups threatened with extinction (Arroyo‐Rodríguez & Dias, 2010; Estrada et al., 2017). Although the effects and modalities of these impacts differ for different species, habitat fragmentation poses a significant threat to their survival (Dirzo et al., 2014; Fahrig, 2003). This threat is often reflected in the behavior of the animal, such as changes in the primate's activity time allocation, roaming distance, and foraging (Campera et al., 2014; Chaves et al., 2012; Chaves & Bica‐Marques, 2013; Irwin, 2008).

Foraging activity is one of the most important behavioral activities for animals to complete (Rayner et al., 2010). Non‐human primates mostly inhabit forest habitats, and, thus, they have developed characteristics that are suitable for forest survival, such as highly flexible eating habits and seasonal use of food and resources (Estrada & Coates‐Estrada, 1996). However, several of these characteristics may influence their ability to live in fragmented forests (Onderdonk & Chapman, 2000). For example, spatiotemporal variations in the availability of food resources may limit the proliferation of certain specialized fruit primates (Estrada & Coates‐Estrada, 1996; Irwin, 2008). Additionally, when the size of the fragments decreases, the forest plant diversity decreases and the vegetation structure degrades (Arroyo‐Rodriguez et al., 2007), which may lead to a decrease in the food supply of the species that inhabit the fragments (Fahrig, 2003; Zanette et al., 2000). Therefore, habitat fragmentation may affect the foraging ecology of species by affecting the habitat quality, such as the abundance, richness, or distribution of food plant resources. The longer habitat fragmentation persists, the greater the differences in the flora composition, vegetation structure, and plant phenological cycles between the forest patches (Arroyo‐Rodriguez & Mandujano, 2006; Hill & Curran, 2003). For example, the abundance of large trees varies between forest fragments (Arroyo‐Rodriguez et al., 2007; Arroyo‐Rodriguez & Mandujano, 2006; Chapman et al., 2007; Dunn et al., 2009; Onderdonk & Chapman, 2000).

However, species do not always passively face changes in habitat and often respond through ecological, behavioral, and genetic adaptations that consist of appropriate adjustments to their survival patterns (Begon et al., 1996). Different species often adopt different foraging and dietary strategies to cope with forest fragmentation and seasonal changes in food distribution and availability. For example, some primates become more flexible in their diet, and some specialized foragers show flexibility in responding to temporal changes in food availability and habitat disturbances (Fahrig, 2003; Hou et al., 2018; Kifle & Bekele, 2021). Furthermore, Johns (1991) found that there were clear differences in the feeding time, food composition, feeding routes, and group patterns between the species in fragmented and non‐fragmented habitats. Generally, the strategies that are adopted by primates to adjust the foraging time allocation can be divided into minimizing and maximizing energy strategies (Schoener, 1971; Zhou et al., 2018). Langurs tend to employ a maximized energy strategy, i.e., spending more time foraging for food to maintain consumption (Zhou et al., 2018; Zhou & Huang, 2021). In addition, the diets of some species differ between intact forests and forest fragments, suggesting that primates exhibit some foraging flexibility (Chaves et al., 2012; Chaves & Bica‐Marques, 2013). This flexible response of primates may include increasing or decreasing dietary diversity, where they consume locally abundant tree species and exotic and secondary successive species, such as vines or climbing plants, or increase their leaf consumption (de Luna et al., 2017; Dias et al., 2014; Irwin, 2008; Onderdonk & Chapman, 2000).

François' langur (*Trachypithecus francois* (de Pousargues, 1898)) is one of the most threatened non‐human primates in the world and is listed as endangered by the International Union for Conservation of Nature (IUCN, 2023). Currently, François' langurs are only distributed in a small area of the karst mountain forests from Vietnam to China, and their distribution is separated by roads, farmlands, and towns into more than 40 independent subpopulations, with a total population of less than 2000 individuals (Zhou et al., 2018). In China, the wild population size is 1589–1718 individuals (Yi et al., 2023). In recent years, habitat fragmentation has had a dramatic impact on the François' langur's population size and behavioral response (Huang et al., 2008; Li et al., 2007, 2009). Although habitat fragmentation has had an overall negative impact on the survival of François' langur, studies of their relatives (*Trachypithecus leucocephalus* Tan, 1957) have shown that they can adapt to habitat fragmentation through shifts in their activity patterns and dietary choices (Huang & Li, 2005; Zhou & Huang, 2021). Given the many differences between the species, rapid habitat changes, and increased human activity, it is critical to improve our understanding of how François' langur has adapted to this fragmented habitat. As previous studies have not focused on some of the highly representative geographical populations, we attempted to explore this aspect.

To better understand the feeding ecology and adaptation to extremely fragmented environments in François' langurs, the diet composition of wild François' langurs was investigated over 10 months in Encheng National Nature Reserve (ECNNR), which includes an extremely fragmented habitat in Southwest Guangxi, China. First, the diets and their seasonal changes were determined. Second, the relationship between food composition and food availability was analyzed, and the feeding strategy of the François' langurs was determined. Finally, the flexibility of the foraging strategy of the langurs was examined. The hypotheses are as follows:

1. Fragments are often characterized by reduced food availability for primates due to changes in forest structure, including the reduction in the number of large trees and increases in the abundance of pioneer species, exotic species, and lianas (Zhu et al., 2004). We hypothesized that François' langurs eat fewer food species and more lianas in extremely fragmented habitats due to lower food diversity.
2. Primates prefer fruits and young leaves and are affected by seasonal fluctuations in food resources (Tsuji et al., 2013; Zhou et al., 2006). Thus, we hypothesized that during the dry season, François' langurs consume fewer young leaves and more alternative type foods, e.g., insects, mature leaves, and flowers, and that the consumption of fruits and young leaves increases with their availability.
3. When preferred foods are scarce, primates usually consume fallback foods (Marshall & Wrangham, 2007). Primates in tropical monsoon forests rely mainly on mature leaves and seeds as fallback foods (Zhang et al., 2017, 2021). Therefore, we predict that the consumption of fallback foods, especially mature leaves, is inversely proportional to the availability of their preferred foods.

## 2 METHODS

### 2.1 Study site and subject

**FIGURE 1:**
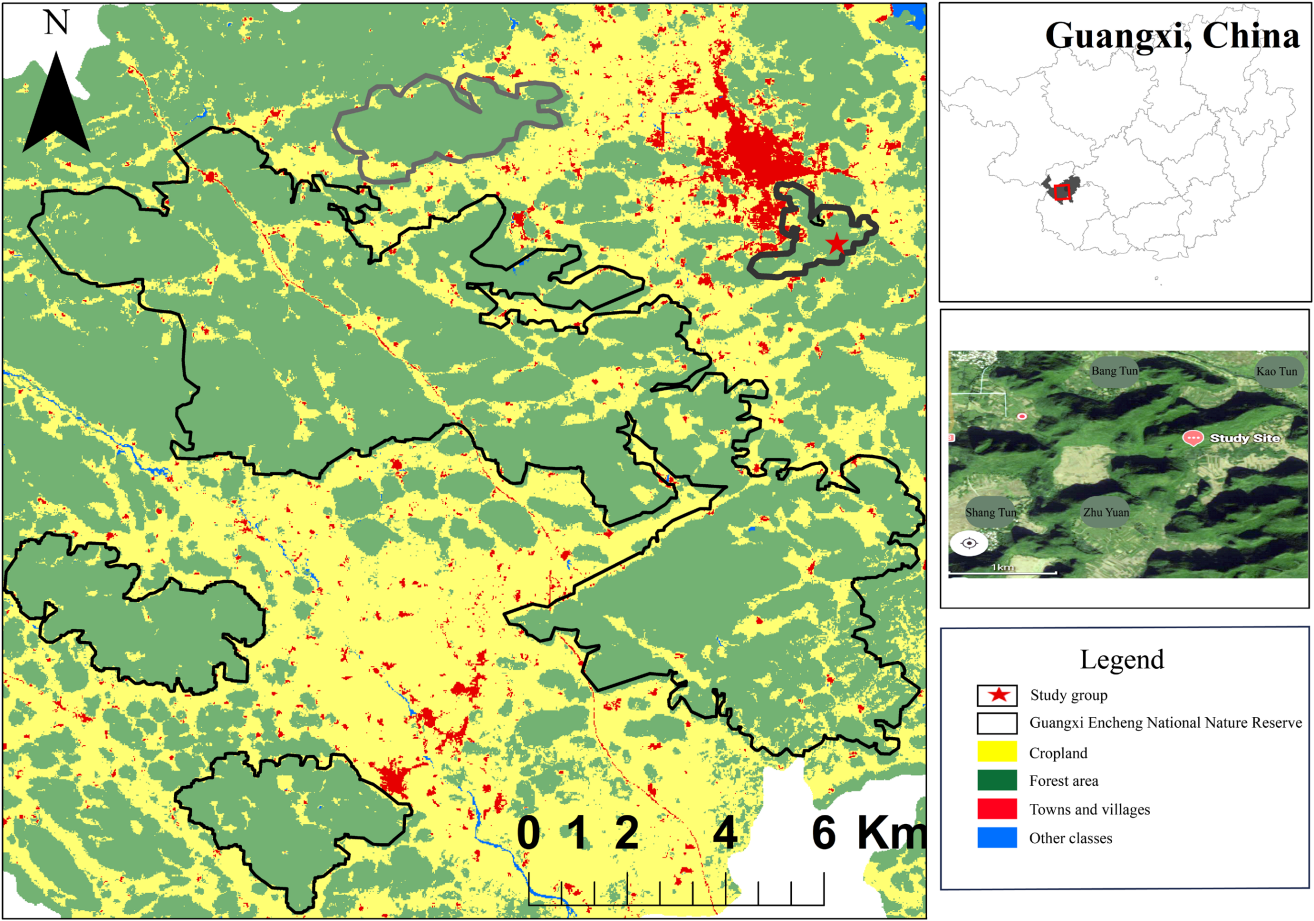
Location of the study site, Encheng National Nature Reserve.

**FIGURE 2:**
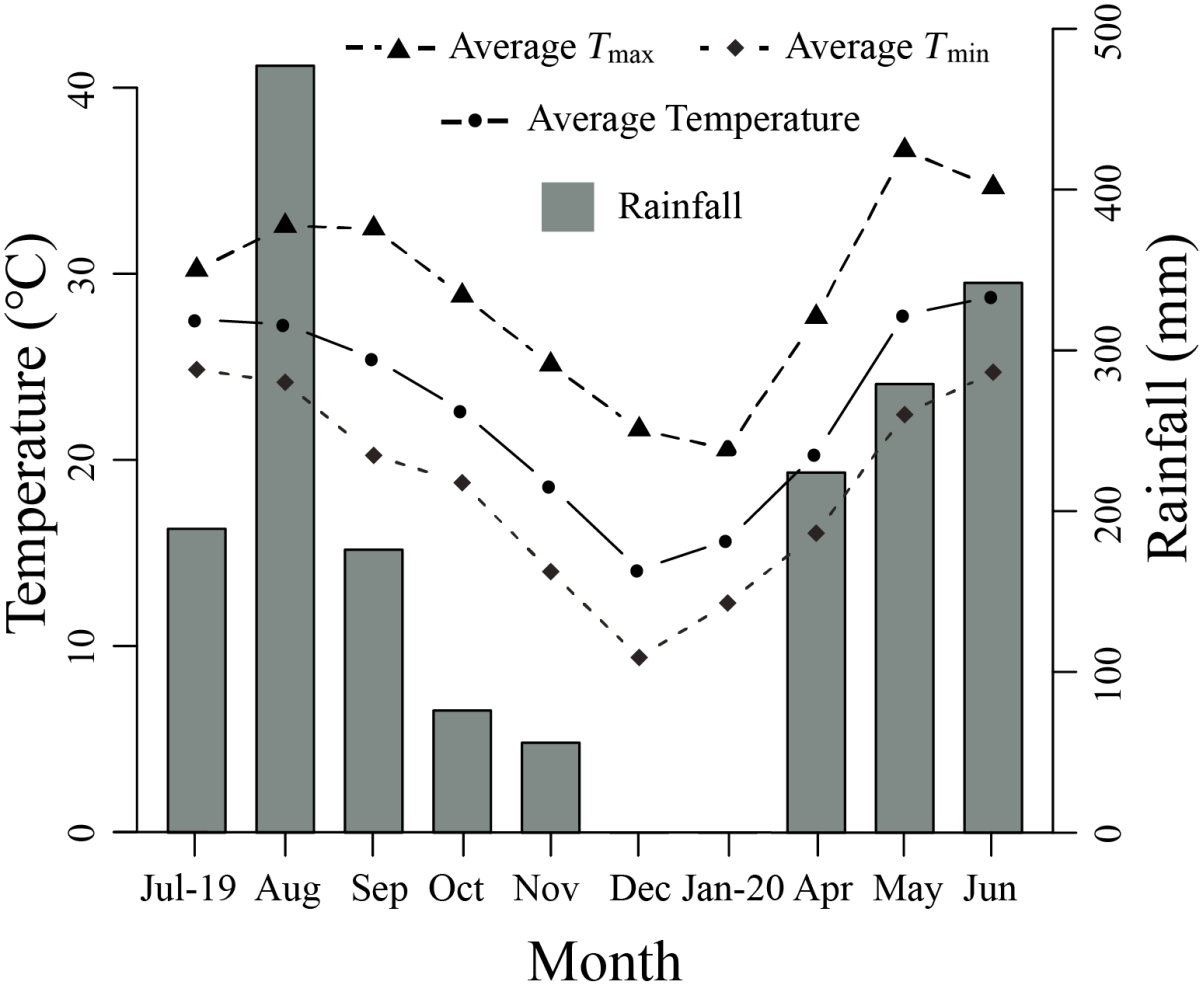
Monthly average temperature (maximum [*T*_max_] and minimum [*T*_min_]) and rainfall at the main study site.

The research was conducted on François' langur at ECNNR, Guangxi Province, China (22°36′32″–22°49′53″ N, 106°58′12″–107°15′45″ E; Figure 1). The main study site was a 258.196 km^2^ karst hill, which is located at an elevation of 300 to 700 m above sea level. From July 2019 to June 2020, the mean minimum and maximum temperatures were approximately 10.22 and 34.28°C, respectively (*n* = 10, the annual average temperature is 22.37°C). The total annual precipitation at the site during the study period was 1462.9 mm, with 83.2% of the rain falling between April and September (Figure 2). There was a dry (October to March) and wet season (April to September) based on the precipitation distribution. One group of François' langur was monitored, which was composed of six individuals (two adult males and four adult females) at the beginning of the study period and increased to eight individuals due to the birth of two infants by the end of the study period.

### 2.2 Vegetation characterization and phenology

The forest in the ECNNR was divided into four habitat types: cliff face, slope, mountain peak, and valley‐floor flatland. These habitats were analyzed using stratified random sampling methods. We sampled 20 plots (20 × 20 m) within the main study site, including four plots that were located in the valley‐floor flatland, nine on slopes, two on cliff faces, and three on mountain peaks. These plots were located in the core area of the langurs' home range, which included more than 4% of the langurs' annual home range (unpublished data). Within these plots, all the trees with a ≥3 cm diameter at breast height (DBH) were tagged. In addition, to evaluate the dominance of each species within a plot, the relative density (RD), relative frequency (RF), and relative coverage (RC) were calculated for species as follows (Burton et al., 2005):

RD = number of individuals of the species *i*/total number of individuals in all the plots;
RF = number of plots with the species *i*/total number of plots;
RC = sum of the basal area of species *i*/sum of the basal areas of all the species.

Based on previous studies of langurs (Zhou et al., 2006) and our two‐month pilot study, 32 plant food species were selected for phenology monitoring. Then, 10 individuals of each species were randomly selected and tagged in the main study area, and a total of 320 trees were monitored. In addition, the data from the vegetation surveys and plant phenology were combined to obtain appropriate estimates of food availability. During the monthly phenology surveys, all the tagged trees were visually inspected for the presence of young leaves, flowers, and fruit, and their abundance (% of crown cover) was scored with a five‐point scale: 0, absent; 1, 0.1%–25%; 2, 25.1%–50%; 3, 50.1%–75%; and 4, 75.1%–100%. The monthly food availability index (FAI) was calculated for the main food items (young leaves, flowers, and fruits) by integrating the density, basal area, and phenology score of the plant species. The formula was as follows:

$$\mathrm{FAI}=\sum_{i=1}^{n}D_{i}B_{i}P_{i},$$

where *D*_*i*_ is the density of the tree species *i* (number of stems/ha), *B*_*i*_ is the average basal area of tree species *i* (m^2^/ha), and *P*_*i*_ is the mean phenology score of the particular food item in the crown of species *i* in a given month (Albert et al., 2013).

### 2.3 Field data collection

Behavioral data of the target langur group were collected for a full day and many other partial days throughout the study. During each full‐day follow, the observations were recorded from when the monkeys left their sleeping sites to when they reentered their sleeping sites at the end of the day. During each partial‐day follow, the data collection began when we first encountered the focal group and ended when they disappeared for over 30 min or entered a sleeping site. The average number of sampling days per month was 8.3 ± 2.19, and the average number of scans per month was 249.20 ± 49.43 (Table S1).

The food items eaten (part and species) were recorded during instantaneous scan samples of 5 min with an interval of 15 min. During each scan, to avoid sampling bias toward certain individuals, the diet data were recorded for as many individuals as possible. If an individual was holding, chewing, or processing a food item, this was considered to be a feeding behavior. Additionally, the plant species that were eaten by the monkeys were recorded via ad libitum sampling; these records were used in a food species list but not for assessing the contributions to the monthly diet.

### 2.4 Data analysis

The percentages of the different plant species in the diet of each study group were calculated using the total feeding records. Similarly, the percentages of the different plant parts in the monthly diet of each study group were calculated using the monthly total feeding records. The Shannon–Wiener index was used to compare the food diversity index (FDI) of the langurs. The formula was as follows:

$$\mathrm{FDI}=H\prime =\sum_{i=1}^{n}P_{i}\mathrm{Ln}P_{i}$$

where H′ is the Shannon–Wiener diversity index, and $P_{i}$ is the percentage of the feeding records of the plant species *i*. Similarly, the diet composition was expressed as the percentage of the feeding time spent on specific food items or food species.

Generalized linear mixed models (GLMMs) were used to examine the influence of season on the diet. Specifically, the number of food species per month and diversity index were tested as the response variables, the seasons were set as fixed factors, and the sample size was set as a random factor. Season was considered a key factor when it influenced the goodness‐of‐fit of the model and the *p*‐value was lower than .05, which indicated a significant difference in the diet between the dry and rainy seasons.

Then, generalized linear models were constructed to examine the influence of ecological factors on the FAI, diet, and dietary diversity. The monthly food availability (including young leaves, flowers, fruits, and mature leaves) was set as the response variable, and climatic factors (including rainfall and temperature) were set as explanatory variables to test the impact of climatic factors on food provision. Similarly, the monthly food species and diversity index was set as the response variable, and food availability (including young leaves, flowers, fruits, and mature leaves), climatic factors (including rainfall and temperature), and diet composition (including young leaves, flowers, fruits, and mature leaves) were set as explanatory variables to examine the influences of diet composition and ecological factors on the langurs' diet. The models considered all possible combinations of all the predictors (total ranked according to their Akaike information criterion [AIC] values). The relative importance of each predictor (Wip) was obtained by summing the Akaike weights for each model. The models with the lowest AIC values were considered to be the top models, and the models within two AIC units (ΔAIC ≤2) of the top models were considered to be highly supported (Burnham & Anderson, 2002). Model‐averaged regression coefficients (*β*) with 95% confidence intervals were used to estimate the effect of each predictor in the models, and the predictors in the highly supported models were determined to be the most important factors affecting the response variables when their 95% confidence intervals for the *β*‐values did not overlap with zero (Xu et al., 2017).

To improve linearity and normality, the numeric variables, such as food availability, were log_10_ (*X* + 1)‐transformed (Xu et al., 2017), and the variables expressed in percentages, such as the feeding time, were log (*X* + 0.00001)‐transformed because the raw data for the nonfood species were zero (Warton & Hui, 2011). In addition, Spearman's rank correlation was used to estimate the relationship among the variables. The normality of all the variables was examined using a one‐sample Kolmogorov–Smirnov test. The GLMMs were performed using the lime4 package in R version 4.0.4 (R Core Team, 2021). The model averaging was performed using the dredge and model.avg function in the MuMIn package (Bartoń, 2019). All the analyses were conducted using R version 4.2.1. All the tests were two‐tailed, with significance levels of .05 (R Core Team, 2023).

## 3 RESULTS

### 3.1 Forest composition and food availability

In the vegetation plots, a total of 150 plant species from 46 families were recorded. The dominance value of the 10 most important tree species ranged from 0.74 to 1.09 (Table 1). The five most dominant species were *Delavaya toxocarpa* Franch., *Ficus virens* Aiton., *Bischofia javanica* Blume, *Pistacia weinmanniifolia* J. Poiss. ex Franch., and *Cipadessa cinerascens* (Pellegr.) Hand. – Mazz. Moreover, the 10 most dominant families were Fabaceae, Lauraceae, Euphorbiaceae, Rhamnaceae, Lauraceae, Rutaceae, Anacardiaceae, Annonaceae, Arecaceae, and Tiliaceae, which accounted for 60.67% of the total stems (range: 2.0%–14.0%).

**TABLE 1:** The top 10 species in Encheng National Nature Reserve, China^a^.

Species	Family	Density (individuals/ha)	Mean DBH (cm)	RD	RF	RC	Dominance	% of stems for family
*Delavaya toxocarpa*	Sapindaceae	983.75	13.62	0.16	0.78	0.15	1.09	33.33
*Ficus virens*	Lauraceae	71.25	12.28	0.01	0.89	0.01	0.91	5.88
*Bischofia javanica*	Euphorbiaceae	47.5	18.09	0.01	0.83	0.01	0.85	5.88
*Pistacia weinmannifolia*	Anacardiaceae	208.75	13.43	0.03	0.78	0.03	0.84	20.00
*Cipadessa cinerascens*	Meliaceae	278.75	13.00	0.05	0.72	0.04	0.80	50.00
*Ficus tinctoria*	Lauraceae	48.75	12.56	0.01	0.78	0.01	0.79	5.88
*Desmos chinensis*	Annonaceae	202.5	9.50	0.03	0.72	0.02	0.78	20.00
*Hainania trichosperma*	Tiliaceae	133.75	10.71	0.02	0.72	0.01	0.76	33.33
*Caesalpinia sinensis*	Fabaceae	463.75	12.52	0.08	0.61	0.06	0.75	4.76
*Flueggea virosa*	Euphorbiaceae	73.75	12.29	0.01	0.72	0.01	0.74	5.88

Abbreviations: RC, Relative coverage; RD, Relative density; RF, Relative frequency.

^a^ Ranked by Dominance.

**FIGURE 3:**
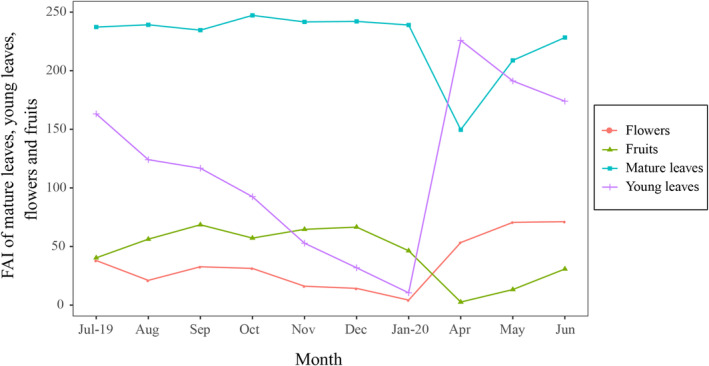
Monthly availability of the mature leaves, young leaves, fruits, and flowers in the main study area in Encheng National Nature Reserve.

The production of young leaves, fruits, and flowers varied considerably among the months (*t*‐test, Young leaves: *t* = 7.299, df = 9, *p* < .01; Mature leaves: *t* = 30.350, df = 9, *p* < .01; Flowers: *t* = 4.800, df = 9, *p* < .01; Fruits: *t* = 5.473, df = 9, *p* < .01). The minimum availability of the mature leaves and fruits occurred in April. The maximum availability of the young leaves, flowers, and fruits occurred during the rainy season, which was April, June, and September, respectively (Figure 3). The phenology also changed significantly between the seasons. In the dry season, the FAI of the young leaves and flowers was significantly lower than that in the rainy season (YL: χ^2^ = 10.79, *p* < .01, FL: χ^2^ = 7.43, *p* < .01). However, there was no significant change in the availability of the fruits and mature leaves (FR: χ^2^ = 2.45, *p* = .12, MF: χ^2^ = 2.10, *p* = .15). Furthermore, a significant positive relationship was documented between the abundance of young leaves and rainfall (*β* = 0.385, Wip = 1.00). Similarly, there was a significant positive relationship between the abundance of flowers and rainfall (*β* = 0.273, Wip = 1.00).

### 3.2 Overall diet and seasonal variations

During the study, the types of food that were consumed by the langurs were divided into two categories: non‐plant parts and plant parts. Among them, the non‐plant parts included cliff minerals and at least one species of insect. The plant parts included 101 identified plant tree species and one nest material. Among the 101 identified tree species, 58.26% were trees (*n* = 67), and 2.61% were herbaceous (*n* = 3), accounting for 66.13% and 1.19% of the foraging records, respectively. Additionally, the langurs consumed 29 species of lianas, accounting for 22.66% of the diet. Furthermore, the langurs consumed two species of parasitic plants, accounting for 7.39% of the diet.

Each month, the langur group consumed between 24 species (April) and 49 species (January), with a monthly average of 38 (standard deviation [SD] 6.64; Table 2). The monthly dietary diversity varied from 2.2 (August) to 3.4 (December), with an average of 3.08 (SD 0.35; Table 2). Additionally, the monthly dietary diversity and the number of food species that were consumed varied seasonally during the study period (species number: χ^2^ = 6.43, *p* < .05; dietary diversity: χ^2^ = 4.10, *p* < .05). The langurs consumed more species and obtained a higher dietary diversity in the dry season than in the rainy season.

**TABLE 2:** Monthly percentages of the feeding records for the different plant parts, number of food species, and food diversity of the study group.

Month	Dietary composition								Number of food species	Food diversity index	Total scan
	Young leaves	Mature leaves	Flowers	Fruits	Seed	Barks	Stems	Others			
Jul‐19	87.2	3.3	2.5	1.7	0.0	0.0	4.6	0.8	39.0	3.3	66
Aug	47.4	0.0	0.8	51.0	0.0	0.0	0.5	0.3	37.0	2.2	93
Sep	64.7	1.6	6.5	21.2	0.0	0.0	5.4	0.5	34.0	3.1	50
Oct	19.8	45.5	4.8	16.6	4.8	1.1	3.7	3.7	40.0	3.1	57
Nov	22.9	42.2	5.5	20.6	3.2	0.0	5.1	0.5	41.0	3.4	56
Dec	21.7	50.2	5.1	13.4	4.7	0.4	4.7	0.0	46.0	3.4	56
Jan‐20	30.6	53.3	1.3	5.9	3.6	2.0	1.0	2.3	49.0	3.4	60
Apr	73.3	0.0	6.2	10.6	0.0	1.9	8.1	0.0	24.0	2.7	45
May	72.4	3.9	1.8	15.9	0.0	0.0	6.0	0.0	36.0	3.2	77
Jun	70.8	0.9	22.3	2.2	0.0	0.0	3.9	0.0	33.0	3.0	70
Annual mean	51.1	20.1	5.7	15.9	1.6	0.5	4.3	0.8	38.0	3.1	63
SD	25.6	24.0	6.2	14.2	2.2	0.8	2.2	1.2	6.6	0.4	14
Dry‐season mean	23.7	47.8	4.2	14.1	4.1	0.9	3.6	1.6	44.0	3.3	57.3
SD	4.7	4.9	1.9	6.2	0.8	0.9	1.8	1.7	4.2	0.2	1.9
Rainy‐season mean	69.3	1.6	6.7	17.1	0.0	0.3	4.7	0.3	34.0	2.9	66.8
SD	13.0	1.7	8.0	18.3	0.0	0.8	2.5	0.3	5.3	0.4	17.7

Fifteen species of plants, including *Ficus concinna* (Miq.) Miq., *Cuscuta chinensis* Lam., and *Boniodendron minus* (Hemsl.) T. C. Chen, were consumed in large quantities throughout the year, accounting for 61.29% of the total records and being the main food of François' langur. Eight species of plants, including *Taxillus sutchuenensis* (Lecomte) Danser, *Henslowia frutescens* Benth., and *Apodytes dimidiate* E.Mey. ex Arn., contributed to a relatively high proportion of the annual feeding records (more than 1%) but were only consumed by the langurs for a few months. For the parasitic vines, the proportion of the annual foraging records was relatively high (1.68%), especially in January, accounting for up to 12.93% of the monthly foraging records. In addition, plants, such as *Ligustrum lucidum* W. T. Aiton and *Cinnamomum burmanni* (Nees & T. Nees) Blume, were only sporadically recorded throughout the year and were often rare.

There was a positive correlation between the consumption of the food species and the mature leaves and the abundance of mature leaves (mature leaves: *β* = 0.018, Wip = 1.00; FAI of mature leaves: *β* = 2.583, Wip = 1.00). There was a negative correlation between the consumption of the food species and flowers and the abundance of fruits (flowers: *β* = −0.107, Wip = 1.00; FAI of fruits: *β* = −0.288, Wip = 1.00). Additionally, there was no significant correlation between the consumption of food species and other dietary composition and ecological factors (Table 3). There was also a significant correlation between the dietary diversity and the mature leaves (mature leaves: *β* = 0.126, Wip = 1.00), and there was no significant correlation between the dietary diversity and the other dietary composition and ecological factors (Table 3).

**TABLE 3:** Effect of diet composition and ecological factors on the number of food species and dietary diversity of the langurs in Encheng National Nature Reserve based on the results of the model averaging.

Variables	Number of month food species				Dietary diversity			
	*β*	2.5%CI	97.5%CI	Wip	*β*	2.5%CI	97.5%CI	Wip
(Intercept)	1.880	1.517	2.242		3.121	2.592	3.650	
Mature leaves	0.018*	**0.001**	**0.035**	**1.00**	0.126*	**0.071**	**0.181**	**1.00**
Young leaves	−0.126	−0.305	0.054	1.00	0.128	−0.514	0.769	0.16
Flowers	−0.107*	**−0.191**	**−0.024**	**1.00**	0.084	−0.261	0.429	0.17
Friuts	−0.048	−0.147	0.051	0.59	−0.135	−0.440	0.171	0.29
(Intercept)	−3.620	−5.949	−1.291		3.844	−0.409	8.098	
FAI (Mature leaves)	2.583*	**1.377**	**3.789**	**1.00**	0.423	−3.891	4.738	0.11
FAI (Young leaves)	0.069	−0.151	0.290	0.53	−1.833	−3.958	0.291	0.75
FAI (Flowers)	−0.111	−0.257	0.035	1.00	1.649	−0.557	3.855	0.87
FAI (Friuts)	−0.288*	**−0.465**	**−0.111**	**1.00**	0.075	−0.640	0.789	0.11
Rainfall	−0.010	−0.081	0.061	0.34	−0.239	−0.779	0.301	0.36
Taverage	−0.247	−0.677	0.182	1.00	−0.575	−4.578	3.427	0.12

*Note*: *β*, model‐averaged regression coefficients; 2.5%CI/97.5%CI, the 95% confidence intervals of regression coefficients *β*; Wip, relative variable importance. Regression coefficients *β* with the 95% confidence intervals excluding zero are denoted by asterisks. Model‐averaged 95% confidence intervals excluded zero listed in bold.

Using all the records in which the food types were confirmed (*n* = 2249), the diet of the study group consisted of slightly more leaves than fruit. Leaves accounted for about 67.7% of the food eaten by the langurs (young leaves 43.9%; mature leaves 23.8%). Fruits and flowers constituted 24.93% of the diet (20.74% fruits and 4.19% flowers), stems and seeds accounted for 6.1% of the diet (4.0 and 2.1%, respectively), and bark and other plant parts constituted 0.24% and 0.97%, respectively (Table 2). Notably, the stems that the langurs consumed were almost all dodder, a parasitic plant (97.9%, 94/96). The langurs liked to feed on all the components of the dodder, especially the stems (Yao Wei, personal observation).

There was significant seasonal variation in the types of foods that the langurs consumed most frequently. They ate significantly more mature leaves and seeds and fewer young leaves in the dry season than in the rainy season (mature leaves: χ^2^ = 4.73, *p* < .05; young leaves: χ^2^ = 22.93, *p* < .01; seeds: χ^2^ = 81.28, *p* < .01). The mature leaf consumption was negatively correlated (*β* = −12.438, Wip = 1.00) with the abundance of young leaves and positively correlated (*β* = 13.501, Wip = 1.00) with the abundance of flowers. The young leaf consumption was positively correlated (*β* = 4.690, Wip = 1.00) with the average monthly temperature, and the flower consumption was positively correlated with the abundance of flowers (*β* = 3.400, Wip = 1.00; Table 4).

**TABLE 4:** Effect of the ecological factors on the diet composition of the langurs in Encheng National Nature Reserve based on the results of the model averaging.

Variables	Mature leaves				Young leaves			
	*β*	2.5%CI	97.5%CI	Wip	*β*	2.5%CI	97.5%CI	Wip
(Intercept)	−33.216	−123.244	56.813		9.304	−6.990	25.598	
FAI (Mature leaves)	21.060	−21.858	63.979	0.76	−6.022	−15.034	2.990	1.00
FAI (Young leaves)	−12.438*	**−24.724**	**−0.152**	**1.00**	−0.026	−0.976	0.925	0.16
FAI (Flowers)	13.501*	**1.456**	**25.536**	**1.00**	−0.069	−0.796	0.658	0.17
FAI (Friuts)	2.010	−7.594	11.614	0.37	0.639	−0.733	2.010	0.75
Rainfall	−0.341	−4.702	4.019	0.13	−0.333	−0.753	0.086	1.000
Taverage	−4.957	−34.836	24.923	0.15	4.690*	**0.753**	**8.627**	**1.000**

Variables	Flowers				Fruits			
	*β*	2.5%CI	97.5%CI	Wip	*β*	2.5%CI	97.5%CI	Wip
(Intercept)	32.473	−6.168	71.113		4.068	−15.365	23.502	
FAI (Mature leaves)	−15.370	−33.849	3.208	1.00	−5.628	−24.707	13.452	0.17
FAI (Young leaves)	−2.463	−5.679	0.753	1.00	−0.066	−0.988	0.856	0.08
FAI (Flowers)	3.400*	**0.545**	**6.254**	**1.00**	−0.243	−1.303	0.816	0.10
FAI (Friuts)	2.574	−0.347	5.495	1.00	0.852	−1.706	3.410	0.26
Rainfall	0.187	−0.715	1.089	0.29	0.383	−0.583	1.350	0.24
Taverage	0.170	−7.447	7.786	0.19	−3.589	−12.224	5.046	0.25

*Note*: *β*, model‐averaged regression coefficients; 2.5%CI/97.5%CI, the 95% confidence intervals of regression coefficients *β*; Wip, relative variable importance. Regression coefficients *β* with the 95% confidence intervals excluding zero are denoted by asterisks. Model‐averaged 95% confidence intervals excluded zero listed in bold.

### 3.3 Food choice

The langurs showed significant selectivity in their diet composition throughout the year. Among the 115 food species (including three non‐plant components and 11 plants whose species names could not be identified) that François' langur consumed throughout the year, only 23 plants (20%) accounted for more than 1% of the total foraging records but contributed to 72.99% of the foraging records. Among the 23 species of plants, *Ficus concinna*, *Cuscuta chinensis*, *Boniodendron minus*, *Pittosporum pulchrum* Gagnep., *Flueggea virosa* (Roxb. ex Willd.) Royle, *Pithecellobium clypearia* (Jack) Benth., *Pericampylus glaucus* (Lam.) Merr., *Vitex kwangsiensis* C. Pei, *Cudrania cochinchinensis* (Lour.) Yakuro Kudo & Masam., and *Erythropalum scandens* Blume were among the top 10, contributing to 49.06% of the foraging records.

The François' langurs did not consume plants within each family evenly. They used 68 (45.3%) of the 150 plant species marked by the vegetation surveys in the overall foraging record, which accounted for 89.04% of the total foraging record (Table 5). Among the top 10 tree species in the vegetation surveys, only *Flueggea virosa* accounted for more than 2% of the total foraging records. The correlation analysis showed that there was no significant correlation between the foraging ratio and relative density of these 68 plants (Spearman Rank Correlation Coefficient *r*s = .146, *n* = 68, *p* > .05), indicating that there was no strong correlation between the François' langur's food choice and the number of plants in the environment.

**TABLE 5:** The top 10 food species, plant parts, and selection ratios for François' langur (*n* = 10 months) from July 2019 to June 2020 in the Encheng National Nature Reserve, China.

Species	Family	Plant type^a^	Parts eaten^b^	Total month used	% of annual feeding time	Relative density	S‐indexd^c^
*Ficus concinna*	Moraceae	T	IF, MF, FR	9	11.78	0.21	2.421
*Cuscuta chinensis*	Convolvulaceae	E	FR, FL, ST	10	5.42	0.02	0.111
*Boniodendron minus*	Sapindaceae	T	IF, MF, FL	7	5.38	0.53	2.873
*Pittosporum pulchrum*	Pittosporaceae	T	IF, MF, FL, FR	9	4.56	1.99	9.081
*Flueggea virosa*	Euphorbiaceae	T	IF, MF, FR	9	3.94	1.21	4.777
*Pithecellobium clypearia*	Mimosaceae	T	IF, MF	9	3.86	2.42	9.356
*Pericampylus glaucus*	Menispermaceae	V	IF, MF	9	3.69	0.18	0.683
*Vitex kwangsiensis*	Verbenaceae	T	IF, MF	7	3.65	2.16	7.882
*Cudrania cochinchinensis*	Moraceae	T	IF, FR	9	3.41	0.31	1.050
*Erythropalum scandens*	Olacaceae	V	IF, MF, ST, FR	9	3.37	0.10	0.346

^a^ Plant type: T, tree; H, herb; V, vine; E, Parasitic plants.

^b^ Parts eaten: YL, young leaves; ML, mature leaves; FL, flowers; FR, fruits.

^c^ S‐index: proportion in annual diet (%) * Relative density (%).

## 4 DISCUSSION

Our results provide new evidence of the underexplored dietary ecology of François' langurs in the fragmented limestone habitat. By documenting the foraging data from unique geographical populations using instantaneous scanning sampling methods, we qualitatively assessed the flexibility and adaptability of François' langurs. The data revealed that the François' langurs' diet has a high degree of flexibility with seasonal variations, which could be advantageous for adapting to current habitat changes. In addition, the François' langurs' diet showed a high degree of selectivity, suggesting that their diet was highly dependent on important species in the habitat.

### 4.1 Dietary characteristics of the fragmented limestone‐living François' langur

Species foraging strategies are closely related to their behavior and ecological plasticity (Chaves & Bica‐Marques, 2013). The same species may adopt different foraging strategies in response to different habitats, which helps them adapt to changes in habitat quality due to different natural or anthropogenic disturbances. In this study, the François' langur ate 112 plant species (including 11 unidentified species). When compared with the two other different geographical populations with similar latitude and climatic conditions, the langurs living in EC NNR fed on more plant species (Nonggang: 92 species; Fusui: 61 species, Table 6), and their diet had a high diversity (3.08). However, when compared to the population in the Mayang River National Nature Reserve, which has a completely different climate, latitude, longitude, and elevation, the langurs in this study fed on fewer species. This result did not support our first hypothesis. The François' langurs at Encheng fed on a larger number of plant species, which was much higher than that of the Fusui group (located in a fragmented habitat) and the Nonggang group (distributed in a more continuous primary forest). Previous studies have shown that there are significant differences in the diet of the different geographical populations of François' langurs, and this difference is likely related to the local vegetation composition (Li et al., 2016). Our results tend to support this view. Although the François' langur lives exclusively in a karst rocky mountain environment, there are large differences in the karst landforms and vegetation composition in different geographical populations. The Guangxi population mainly inhabits the northern tropical monsoon forest at an elevation of 300–600 m, and the landform is dominated by peaks and depressions, whereas the population in Guizhou mainly inhabits a subtropical evergreen broad‐leaved forest and coniferous and broad‐leaved mixed forest at 600–1800 m, and the landform is dominated by peaks and valleys. Therefore, the differences in the feeder plant species in the different geographic populations of François' langurs also reflect the flexibility of their behavior that enables them to adapt to different habitats.

**TABLE 6:** The number of plant species in the different sites that were consumed by the four geographic populations of François' langur.

Province	Site	Number	Lifestyle				Diversity	Landscapes	Vegetation	Origin
			T	V	H	E				
Guangxi	NG	92	38	52	1	1	2.48	C	T	Huang et al. (2010)
Guangxi	FS	61	42	16	3	0	3.03	F	T	Li et al. (2015)
Guangxi	EC	112	67	29	3	2	3.08	F	T	This study
Guizhou	MYH	129	99	23	7	0	—	C	S	Hu (2007)

*Note*: NG: Nonggang NNR; FS: Fusui NNR; EC: Encheng NNR. MYH: Mayang River NNR; Landscapes, F: Fragmented limestone habitats; C: Continuous limestone habitats. Vegetation, T: Tropical monsoon forests; S: Subtropical evergreen broad‐leaved forests and mixed coniferous and broad‐leaved forests.

High‐quality habitat often means more food variety but it can also result in a high abundance of a single plant species. Animals can adjust their diet according to their food preferences and consume more of their preferred plant species. This has been confirmed in other studies of colobus monkeys, where only some of the species are consumed despite the high diversity of the plant species in the forests (Bang et al., 2019; Kuladeep et al., 2011; Le et al., 2019; Workman, 2010). Similar conclusions have been found for other langurs; for instance, white‐headed langurs (*Trachypithecus leucocephalus* Tan, 1957) selectively use some plants in their habitat (Zhang et al., 2017). However, when the number of preferred foods is insufficient, monkeys can expand their diets and eat more alternative foods to overcome the lack of their preferred foods (Zhou & Huang, 2021). The high level of food diversity in the ECNNR may also be evidence of this strategy. According to the vegetation survey, the diversity of the plant species in the study area reached 186 species/ha (DBH ≥3 cm). This is much larger than the abundance in Fusui of 70 species/ha (DBH ≥1.2 cm; Li et al., 2015). Thus, even though the habitats are extremely fragmented, many of the fragmented habitats may act as safe havens, where relatively rich vegetation conditions are preserved locally. This provides an adequate food source for the langurs. This suggests that François' langurs not only have a wide dietary composition but can also adjust their dietary composition according to the differences in the plant species in the habitat, and foraging flexibility is one of the important reasons for their survival in various habitats (Chaves & Bica‐Marques, 2013; Garber et al., 2009).

Consistent with other studies, the langurs in Encheng fed primarily on leaves, especially young leaves. According to the theory of optimal foraging, animals have broad‐spectrum or specialized diets (MacArthur & Pianka, 1966). The langur's unique digestive system, digestive organs, and teeth make it better suited to fermenting and utilizing cellulose‐rich foods, such as leaves, than any other primate, apart from Colobus monkeys (Zhou et al., 2018). Overall, the leaf feeding of the langurs in Encheng was the same as that of another fragmented population, the Fusui group. However, the langurs in Encheng fed on fewer young leaves than those in Fusui (Table 7). These differences may be related to differences in the vegetation structure of the habitat. In fragmented habitats, the absence of tall trees stimulates plant growth and produces large numbers of young leaves (Ganzhorn, 1995), which provides a stable food source for François' langurs in fragmented habitats. However, the small size of the area may limit the feeding range of the langurs, and vegetation that is close to farmland is often avoided (personal observation). Therefore, increasing the intake of mature leaves has become an effective means to solve this problem. In addition, the langurs also fed on more fruits in Encheng than those in the other two populations in Guangxi, which may be related to the larger number of *Ficus macrophylla* in the distribution area, which are often considered to be a high‐quality source of sugar and water (Zhou et al., 2018). Thus, in addition to changing their diets, François' langurs responded to differences in their environment by eating different parts of the plants.

**TABLE 7:** The number of food composition in the different sites that were consumed by the four geographic populations of François' langur.

Province	Site	Dietary composition (%)									Origin
		L	YL	ML	FR	FL	SD	ST	P	O	
Guangxi	NG	71.0	46.9	24.1	13.2	6.3	4.3	1.8	1.0	2.5	Huang et al. (2010)
Guangxi	FS	67.9	55.8	12.1	12.4	4.3	10.1	0	0	5.3	Li et al. (2015)
Guangxi	EC	67.7	43.9	23.8	20.7	4.2	2.1	4	0	1.0	This study
Guizhou	MYH	63.9	43.3	20.6	25.7	1.1	6.5	—	—	2.8	Hu (2007)

Abbreviations: E, Parasitism; EC, Encheng NNR; FL, Flowers; FR, Fruits; FS, Fusui NNR; H, Herb; L, leaves; ML, Mature leaves; MYH, Mayanghe NNR; NG, Nonggang NNR; O, Other; P, Petiole; SD, Seed; ST, Stem; T, Tree; V, Lianas; YL, Young leaves.

### 4.2 Dietary response to phenology change

The quantity and quality of food resources are not always evenly distributed in space and time (Fryxell, 1991). Seasonal changes in temperature and precipitation often result in changes in primate foraging strategies by altering the availability of food resources (Oates, 1987). Our study site belongs to the typical northern tropical monsoon forest climate zone, which has a distinct dry and rainy season. The high temperatures and rainfall of the rainy season tend to lead to abundant food, while the dry season with little rain causes severe food shortages. In this study, the langur expanded its food intake during the dry season (dry season: 44 species; rainy season: 34 species) and increased its food diversity (dry season: 3.3; rainy season: 2.9). This effectively relieved the foraging pressure caused by food scarcity. This finding is consistent with other studies (Li et al., 2003). For instance, François' langurs increased the number of foraging plant species during the dry season to cope with the lack of their preferred food in the Nonggang area of Guangxi (Zhou et al., 2006). Additionally, in the Chongzuo region of Guangxi, white‐headed langurs increased their intake of plant species during the dry season (Zhou & Huang, 2021), while in Malaysia, Presbytis femoralis fed on more types of food during the food shortage season (Bennett, 1983). This trait was also seen in other food‐eating primates, such as Lophocebus albigena, which increased their food diversity during the months when the fruit abundance was lower (John et al., 2001). These studies all supported the suggestion that primates can survive periods of food scarcity by adjusting the food diversity of their diet.

François' langurs also survive long dry seasons by adjusting their food composition (Zhou et al., 2018). The results of this study showed that the François' langurs increased their feeding on mature leaves when the availability of young leaves decreased during the dry season, and most of the seed and bark records occurred in the dry season (seed: 100%, bark: 85.6%). Moreover, the number of food species and food diversity index of the François' langurs increased with the increase in the availability of mature leaves. The number of food species decreased with the increase in the availability of fruits and the proportion of flowers. This is thought to be because these variables enable the langurs to adjust their food composition to respond to climate and habitat changes. This was supported by other studies. According to Zhou et al. (2006), during the dry season when the food was scarce, the Nonggang François' langur consumed mature and young leaves as an alternative food to solely young leaves. In Fusui, Li et al. (2009) found that François' langurs also increased their intake of mature leaves in the dry season. In Chongzuo, Guangxi, white‐headed langurs fed on more young leaves during the dry season but increased their intake of mature leaves (Li & Rogers, 2006). Moreover, the long‐tailed langur (*Semnopithecus entellus* (Dufresne, 1797)) in the Kanha region of India will eat mature leaves in large quantities when there is a shortage of young leaves, fruits, and flowers (Newton, 1992). Therefore, this is thought to be one of the ways that langurs can adjust their food composition to respond to climate and habitat changes and maintain their energy intake.

### 4.3 Food choice

Previous studies have shown that primates are selective about the types and components of their food, including the selection of plant species and food portions (Bennett, 1983; Newton, 1992; Workman, 2010; Zhou et al., 2018). This selectivity is influenced by factors, such as the quality and quantity of food (e.g., nutrients, cellulose, mineral elements, secondary toxicants, and food availability) and water content (Zhou & Huang, 2021). The findings of this study were consistent with previous studies, and the Encheng François' langur had obvious food selectivity. Moreover, food selection is not strictly based on the number of plants in the habitat. In this study, 72.99% of the total foraging records of the François' langurs consisted of 23 plant species. They consumed 68 of the 150 plant species that were identified in the vegetation surveys based on the overall foraging record, which accounted for 89.04% of the total foraging record. The top 10 tree species in the vegetation survey included *Pittosporum pulchrum* and *Vitex kwangsiensis*, which accounted for more than 2% of the total foraging records. This was similar to the findings of other studies. François' langurs showed selectivity in the plant species they ate, and the 10 plants that were most consumed accounted for 51%–90% of the food composition in the other geographic populations (Zhou & Huang, 2021). In summary, François' langurs living in the ECNNR have similar feeding behaviors to other primates, their choice of food is independent of the number of plant species in the habitat, and the most common tree species in the habitat are only consumed in a small amount.

Overall, we found the François' langurs in the fragmented limestone forest in southwest China had a similar feeding ecology to other groups in distinct habitats. However, there were also some differences. We speculate that these dietary differences are mainly due to environmental conditions. Furthermore, although our results indicate that François' langurs respond to limestone habitat fragmentation by broadening their diet and relying more heavily on leaves, their core food source is still provided by a small number of plants. Therefore, we also emphasize the importance of conserving uncommon plant species and maintaining existing habitats for the survival of the langurs. Future management efforts should aim to increase the collection of foraging data for more populations and improve foodborne plant cultivation plan based on langur recipes.

## AUTHOR CONTRIBUTIONS

**Wei Yao:** Data curation (equal); investigation (equal); writing – original draft (equal). **Cheng‐Ming Huang:** Writing – original draft (equal). **Jia‐Xin Zhao:** Investigation (equal). **Rong Huang:** Investigation (equal). **Wen‐Hua Li:** Investigation (equal). **Peng‐Lai Fan:** Project administration (equal); supervision (equal). **Qi‐Hai Zhou:** Funding acquisition (equal); project administration (equal).

## CONFLICT OF INTEREST STATEMENT

The authors declare that they have no known competing financial interests or personal relationships that could have appeared to influence the work reported in this paper.

### OPEN RESEARCH BADGES

This article has earned Open Data, Open Materials and Preregistered Research Design badges. Data, materials and the preregistered design and analysis plan are available at https://doi.org/10.5281/zenodo.10597573.

## Supporting information


Table S1.

## Data Availability

Raw data that support the findings of this study will be available on the DYAD. Relevant data files are available on Dryad at https://datadryad.org/stash/share/t7QfHW2goo_LZysxagJV230OV‐dBxuzqkCdZW32uTgU. The associated DOI will be https://doi.org/10.5061/dryad.2rbnzs7wb.
