# Supplementary material for: Feeding adaptation of François' langurs (Trachypithecus francoisi) to the fragmented limestone habitats in Southwest China
Source: Ecol Evol. 2024 Apr 22;14(4):e11269. doi: 10.1002/ece3.11269 (PMC11035973; doi:10.1002/ece3.11269)
Supplement: Supplementary file 1 — Table S1. [file ECE3-14-e11269-s001.docx]

**Appendix S1**

**TABLE S1 Number of behavioral samples**

| Month | Total days | Total scan | Scan of diet | Scan of individuals |
| --- | --- | --- | --- | --- |
| Jul-19 | 7 | 189 | 66 | 840 |
| Aug | 11 | 327 | 93 | 1442 |
| Sep | 7 | 230 | 50 | 853 |
| Oct | 9 | 257 | 57 | 866 |
| Nov | 8 | 243 | 56 | 888 |
| Dec | 6 | 196 | 56 | 921 |
| Jan-20 | 6 | 225 | 60 | 1072 |
| Apr | 6 | 211 | 45 | 749 |
| May | 11 | 295 | 77 | 1190 |
| Jun | 12 | 319 | 70 | 1073 |
| Annual mean | 8.30 | 249.20 | 630 | 989.40 |
| SD | 2.19 | 49.43 | 49.43 | 207.58 |
| Dry-season mean | 7.25 | 230.25 | 57.25 | 936.75 |
| SD | 1.30 | 26.32 | 1.89 | 92.96 |
| Rainy-season mean | 9.00 | 261.83 | 66.83 | 1024.50 |
| SD | 1.92 | 59.19 | 17.66 | 262.07 |
